# Native Chemical Computation. A Generic Application of Oscillating Chemistry Illustrated With the Belousov-Zhabotinsky Reaction. A Review

**DOI:** 10.3389/fchem.2021.611120

**Published:** 2021-05-11

**Authors:** Marta Dueñas-Díez, Juan Pérez-Mercader

**Affiliations:** ^1^Department of Earth and Planetary Sciences and Origins of Life Initiative, Harvard University, Cambridge, MA, United States; ^2^Repsol Technology Lab, Madrid, Spain; ^3^Santa Fe Institute, Santa Fe, NM, United States

**Keywords:** oscillatory chemical systems, chemical computing languages, computing automata, thermodynamics of computation, Turing machine, maximum entropy principle, chemical computing paradigm, Belousov-Zhabotinsky oscillatory reaction

## Abstract

Computing with molecules is at the center of complex natural phenomena, where the information contained in ordered sequences of molecules is used to implement functionalities of synthesized materials or to interpret the environment, as in Biology. This uses large macromolecules and the hindsight of billions of years of natural evolution. But, can one implement computation with small molecules? If so, at what levels in the hierarchy of computing complexity? We review here recent work in this area establishing that all physically realizable computing automata, from Finite Automata (FA) (such as logic gates) to the Linearly Bound Automaton (LBA, a Turing Machine with a finite tape) can be represented/assembled/built in the laboratory using oscillatory chemical reactions. We examine and discuss in depth the fundamental issues involved in this form of computation exclusively done by molecules. We illustrate their implementation with the example of a programmable finite tape Turing machine which using the Belousov-Zhabotinsky oscillatory chemistry is capable of recognizing words in a Context Sensitive Language and rejecting words outside the language. We offer a new interpretation of the recognition of a sequence of chemicals representing words in the machine's language as an illustration of the “Maximum Entropy Production Principle” and concluding that word recognition by the Belousov-Zhabotinsky Turing machine is equivalent to extremal entropy production by the automaton. We end by offering some suggestions to apply the above to problems in computing, polymerization chemistry, and other fields of science.

## Introduction. What Is a Computation? and How Does Chemistry Do It?

Computation is about information and its transformation. Given (Evans, 2011) some input information during a computation this information is suitably transformed and output in a way that can be used for some functional purpose. We can input into some machine the number of hours worked by employees, input information for an hourly rate and, if so instructed, the machine will give the dollar amount for each employee which is then communicated to payroll … which, hopefully, will issue a funded paycheck! In a general sense a computation consists of the (a) input, (b) transformation, and (c) output of information that can then be used for some useful purpose (Feynman, 2000; Evans, 2011). For the above to take place, one needs to have some available information and a means of transforming it into some different form which is then output.

The “transformation” of the information must be (Feynman, 2000) “mechanical,” i.e., same input information gives same output information. “Information” above is understood as “what is conveyed or represented by a particular arrangement or sequence of things” (Simpson and Weiner, 1994). The “things” can be any collection of objects, letters, numbers, or shapes, to mention just a few instances. Information, therefore, can be encoded into a sequence of symbols.

Let us now consider the general features of chemical reactions (Pauling, 1947, pp. 12, 95) in parallel to the previous description of a computation. In a chemical reaction, substances in the correct proportions get together, react according to a reaction mechanism, and generate a particular output. We can think of the reactants and the sequence in which they are brought into the reaction as input information. If we are at a scale where we can ignore the effects of fluctuations, we can think of the reaction mechanism as a means for mechanically transforming the reactants into some output products. We can immediately see that there is a clear parallel between a computation and a chemical reaction.

Therefore, in principle we can establish an analogy between chemistry and computation and unlock the power of chemistry to do some form of computational work. This, however, requires that we understand how to represent information in a form that can be (mechanically) processed by chemistry.

**Figure 1:**
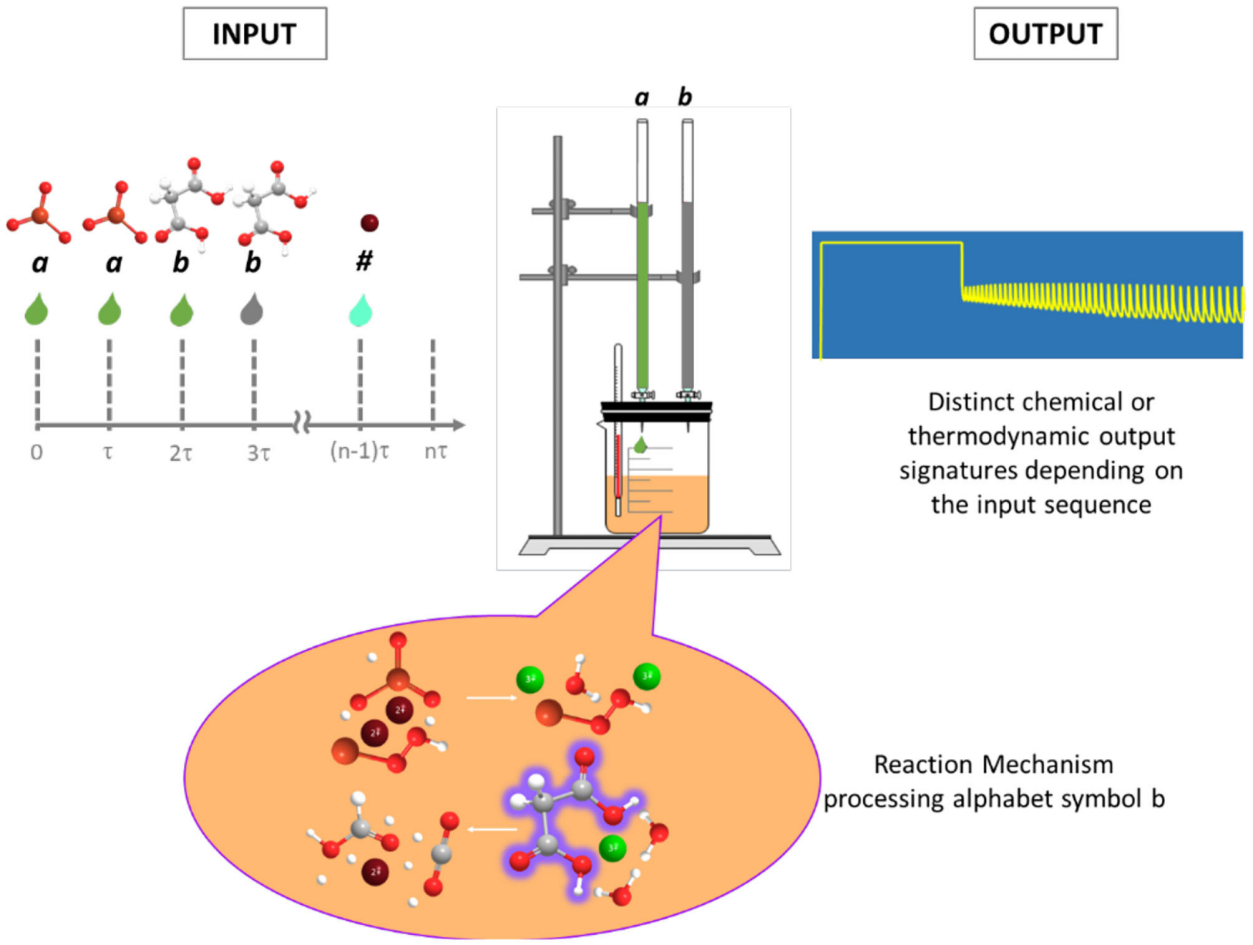
Schematic representation of native chemical computation. Each alphabet symbol is assigned a corresponding reactant, and the sequence of aliquots of these symbols represent the input word to be processed. The processing is carried out autonomously by the reaction mechanism and produces a distinct chemical signature that depends on the sequence and represents the output of computation (adapted from Figure 1 in Dueñas-Díez and Pérez-Mercader, 2019a).

In chemistry, information resides in the relative positions of atoms in molecules, in the bonds, electron densities, and a long etc. of the properties of molecules and atoms (Sienko and Lehn, 2003). We can also take inspiration from natural life and represent information using chemistry following the way in which genetic information is represented with DNA: by means of an alphabet constructed with the purine and pyrimidine molecular bases, arranged in a certain order to represent and carry information which is (mechanically, i.e., systematically) processed in the cell's ribosomes. By analogy we can think of using a sequence of chemicals as a means to represent information with chemistry. The chemicals in the sequence could be the reactants for some reaction; the chemical transcription of the symbols would consist of the assignment of specific aliquots of the reactants to each symbol. Then the sequence in which the aliquots are fed to the reaction would represent the chemical transcription of the original symbolic sequence (cf. Figure 1).

The various aliquots of the reactants could represent “letters” in an abstract alphabet and some of their ordered sequential groupings would represent “words” in a language which is generated by an underlying grammar. If the aliquots correspond to chemical substances that react, we then have a parallel between letters/symbols, words in a language, and a “mechanical” processor of their information which is the chemical reaction (and the pathways contained in its mechanism). The products of the reaction in quantity and quality (including radicals) and the corresponding values of the state functions of the chemical system after it reaches some stable regime (not necessarily equilibrium) are the (chemically autonomous) material and physico-chemical “result” of the computation! We call this form of computation “native chemical computation” to emphasize the fact that it is only unassisted chemistry carrying out all the computation autonomously once the information has been input to the “machine.” All the computation is done by molecules and atoms participating in the reaction. Note that “chemical computation” is a very broad topic and there are different approaches, most of them making use of reaction-diffusion, geometrical aids, and other types of hybrid approaches (Tóth and Showalter, 1995; Adamatzky and Costello, 2002; Gorecki et al., 2003; Ross et al., 2006; Wang et al., 2016). For an excellent review see Adamatzky (2019). Another interesting form of chemical computation uses chemical reactions to implement Fuzzy logic (a form of logic that deals with approximate modes of reasoning) and provides an approach to applications of chemical computation related to those of Fuzzy logic (Gentili, 2018 and references therein). Here, however, our focus is strictly on the “Native Chemical Computation” approach as defined above (In the remainder of the text whenever we talk of chemical computation we thus refer to Native Chemical Computation).

A chemical computation can involve of the order of Avogadro's number of molecules all of which contribute to the computation taking place in the reactor. This is indeed a very large level of parallelism but, unfortunately, it is offset by the huge level of correlation and synchronization of the molecules, so that effectively there seems to be a waste of the potentially available computational power in the molecules. In this paper, we will review some steps taken recently in the direction of harnessing this “waste” by making it more efficient.

After this brief Introduction, we will continue with a discussion of “How computations are performed,” followed by a discussion of “What chemistry brings to computing;” these will be followed by sections devoted to “How Chemistry Computes,” to a discussion of “Oscillatory Chemistry and Native Chemical Computation” then there is a section devoted to “The Interpretation of word acceptance in a native chemical Turing Machine” and on to a final section covering the necessary details for “Building a Turing machine using the BZ reaction Chemistry.” We finalize with a brief Discussion and Conclusions section. The review is complemented by a basic bibliography where further references can be found, a list of Nomenclature and Definitions used in the review and four more technical Appendices.

## How Computations Are Performed

We can imagine a computation (in a mathematical sense) as the application of a sequential series of operations, or procedure, on some data which are carried out in order to solve some mathematical problem. For example, to solve a differential equation describing the time evolution of some substance participating in a chemical reaction. Such a systematic procedure is called an algorithm, and the execution of an algorithm by a human “computer” was essentially the way in which Turing (1936) envisaged the “mechanization” of a computation back in the 1930s. The result of these studies was the introduction of an automaton^1^ capable of implementing any algorithm, even though the automaton might take a very long time to solve the problem. The class of automata capable of doing any computation constitute the class of “Turing Machine” automata. In this latter class there are automata which require an infinitely long tape and are therefore not accessible to representations with real material components, which can only use a bounded amount of energy and translates into a class of finite tape-length automata (Minsky, 1967; Linz, 2012). In what follows, we will restrict ourselves to automata which do not require any infinitely long tapes or unbounded amounts of energy for their operation.

The idea behind these automata is that a mathematical problem involving computations can be reduced to a (perhaps very large, but always finite) number of steps involving linear and non-linear (Sheffer, 1913; Lloyd, 1992) operations. The linear and non-linear operations can be performed using various approaches: analogical and digital, sequential or in parallel, and combinations thereof. The computing machinery can be of many types: geometrical, mechanical, electronic, or chemical.

In the case of analog computation numbers are represented by some continuous property, for example, lengths in a slide rule (Stoll, 2006) or chemical concentrations, and we measure another length to obtain the “result.” For example, in the case of the slide rule, there is an analogy (or correspondence) between adding lengths and the addition of the logarithm of numbers. In this way we can represent the basic arithmetic operations on numerical quantities by analogy: we input some continuous physical quantities and measuring another (analog) physical quantity gives us the result of some operation. Analog computation depends specifically on the analogy between a physical property and its mathematical formulation, which therefore limits the application of an analog machine. Another incarnation of analog computation considers a particular physical phenomenon and the differential equations that represent it. Then, by physically implementing one instance of the phenomenon whose differential equations are known one can, in principle, establish an analogy with any other phenomenon that follows the same differential equation. A standard example of this is the relationship between RCL electrical circuits and damped springs (Truitt and Rogers, 1960).

Alternatively, we could operate directly with numbers (digits) in the same way we do with pencil and paper, and therefore generate an approach that can be used to tackle any problem solvable by arithmetic procedures. Digital computation is of course closely connected to Boolean logic and the immensely important (unpublished) 1880's work by Peirce on the functional completeness of the NOR Boolean function (see Nahin, 2013) and the completely independent 1913 Sheffer's (Sheffer, 1913) result that the NAND Boolean function is also functionally complete (i.e., any of the other Boolean functions can be represented with combinations of the NAND or the NOR gates). This together with the representation of data using a binary system, the fast switching capabilities available with electrons in electronic devices, especially solid state, and the direct electrical interconnectability of logic gates are some of the fundamental bases for today's dominance of electronic computers. Indeed, using this approach one can carry out both linear and non-linear operations in general and, sometimes, also energetically efficiently.

But there are many other equivalent possibilities for digital computing. For example (Lloyd, 1992), if one has only gates that produce a constant output, that multiply their input by constants, plus gates that add these inputs together and which can be interconnected by wires and fanouts then one can carry out any linear operation. However, to be able to do general computation which involves both linear and non-linear operations, one needs to supplement the above with a means to perform non-linear operations. Indeed, it can be seen that any non-linearity on top of the above linear gates is sufficient to enable non-linear general computation (Lloyd, 1992). That is, the presence of a switch is a fundamental enabler of general computation.

In summary then, to carry out a general digital computation using logic gates we need input information which must be fed in a timely fashion to some appropriate array of linear gates, non-linear switches and fan outs and, finally, in the end presented as the result of the computation. In other words, if we are computing with gates we will need to be able to transport information from the location of the output of a gate at one particular spatial location, to another location where the input to the next gate is located. The process of switching and feeding of the digital sequence to the gates must be timed very precisely. This information transportation process requires that the traveling information be unaffected during the actual travel of the information carriers (electrons in electronic computing). Otherwise, noise will reduce the accuracy of the computation, perhaps to intolerable levels.

For any interesting computation this implies that the information must be shuttled between interconnected gates and the various events and their timing carefully synchronized. Except because of thermal dissipations this is, in general, not a problem for electronic computers, where the digital information (written in 0 and 1 s) is carried by electrons moving at speeds close to 30,000 km/s, and can be directed by means of cables and multiplexing circuits. This state of affairs of course changes dramatically when one needs to deal with millions of gates and the computation is not reversible (Landauer, 1961).

Unfortunately, when using gates for chemical computation this becomes a serious problem because the shuttling of information by mass transport necessarily involves molecular transport by diffusion, convection, or molecular migration all of which degrade information. For example, diffusion in chemistry not only brings into play the “Arithmetic demon” (Ireland, 1969; Serratosa, 1990; Gilbert and Martin, 2016) of chemistry hindering conversion, selectivity, and reaction yields but diffusion, *per se*, increases entropy and consequently efficiently and irreversibly information is destroyed as it travels and time elapses (Similar statements can be made about convection or unguided molecular migration). Separately or together due to the rapid and prohibitive accumulation of errors during these processes, the above makes very difficult (if not quickly impossible) the use of gates when both the information carrier and the information processing machine are chemical in nature. This makes the strategy of using gates in native chemical computation even more difficult if one intends to use chemistry for relevant computations and information processing at any level of meaningful complexity.

However, not all is lost, as there is another way to solve computational problems and implement the mechanics of the computation required by the algorithm which does not require the use of arrays of linked Boolean logic gates. Other automata exist in computer science which are more advanced than gates and are capable of performing the computational equivalent of many gates together and classifying the results of the input information as either Accepted or Rejected (Adamatzky and Costello, 2002; Rich, 2008). This provides a powerful workaround to the problems with gates in chemistry and enables chemical computation in a framework where chemistry is both carrier and processor of the information. Interestingly, the resulting computing architecture inextricably integrates both processor and memory via the chemical reaction (i.e., it is a non-von Neumann architecture).

## Computing With Automata

As mentioned in the Introduction, a basic element in a computation is the matching of information, just as it happens with molecules in a chemical reaction when under certain conditions some molecules react (the information in the reacting molecules matches) or do not react (the information in the molecules does not match). The fact that anything can be represented by a string of symbols is a very powerful concept and it is because of this that sequence recognition can be naturally translated into chemistry. Of course, this requires a particular encoding of the object (the “thing”) using symbols belonging to an alphabet. And to be successful one needs to encode the problem to be solved we wish to solve as a string which would then be presented as a “word” in some language that an automaton could recognize as belonging to this language. At first sight the above sounds like an intractable problem: there could be so many languages! But today the important question of defining the right language for a problem is a standard task in Computer Science and is discussed in many modern textbooks (e.g., Sudkamp, 2006; Rich, 2008).

Work on languages and computation done during the 1950s and 1960s proved the remarkable fact that all languages could be classified according to their complexity into a four-level hierarchy of languages. This hierarchy of languages is known as the Chomsky Hierarchy of Languages (Chomsky, 1956) after the linguist Norman Chomsky who proposed it and made major contributions to its development. The hierarchy is shown and described in Table 1 (cf. Dueñas-Díez and Pérez-Mercader, 2019a, 2020). Equally remarkably, accompanying this classification and hierarchy of languages, there is a parallel hierarchy of automata which have the appropriate structures to identify one or more languages at their level in the hierarchy and languages at all the levels below its own. Because of this, the hierarchy of automata and languages is inclusive, which is a fundamental property of the hierarchy and has deep implications for programmability. Furthermore, languages and automata go up in complexity as we go to higher orders in the hierarchy. That is members at lower levels are simpler constructs.

**Table 1:** The Chomsky hierarchy.

**Grammars**	**Languages**	**Accepting automata**
Type 0 grammars, phase-structure grammars, unrestricted grammars	Recursively enumerable	Turing machine, non-deterministic Turing machine
Type 1 grammars, context-sensitive grammars	Context-sensitive	Linear-bounded automata (bounded tape-length Turing machine)
Type 2 grammars, context-free grammars	Context-free	1-Stack pushdown automata
Type 3 grammars, regular grammars, left-linear grammars, right-linear grammars	Regular	Deterministic finite automata, non-deterministic finite automata

*Languages are generated by grammars. By gradually imposing restrictions on them (Hopcroft et al., 2007), grammars are categorized into an inclusive four level hierarchy, the Chomsky hierarchy. Type-0 are unrestricted grammars and Type-3 the most restricted. Type-1 grammars correspond to Type-1 Languages which are also called Context Sensitive Languages (CSL). Type-2 and Type-3 correspond to Context Free (CFL) and Regular (RL) languages, respectively. Each class of languages in the Chomsky hierarchy has been characterized as the languages generated by a family of grammars and accepted by a type of machine. The relationships developed between generation and recognition are summarized in this table which is adapted from p. 338 of Sudkamp (2006). Type 0 and Type 1 grammars are accepted by Turing machines, hence the horizontal line in the Table that separates them from Types 2 and 3. Reprinted from Dueñas-Díez and Pérez-Mercader (2019a) Copyright (2019) with permission from Cell Press*.

The simplest languages are called “Regular Languages” and they are accepted also by the simplest automata, the Finite Automata (FA) class which includes all the Boolean logic gates. These automata do not have memory. The FA can be extended by incorporating in them a memory of the last-in-first-out type (LIFO) so that the automaton can store and retrieve information. This memory is called the stack. The class of FA endowed with the rules necessary to operate the memory are called the 1-stack Push Down Automata (1-stack PDA) because they have one memory stack of the LIFO type: information can be stored by putting it at the top of the stack while pushing down the stack any previously stored information items.

So, one may ask, can we give more power to the 1-stack PDA by adding more stacks? The answer is yes, but surprisingly 2-stacks is all one needs in order to achieve maximum physical computing power (Hopcroft et al., 2007). That is, these systems will be limited in their computational power by the amount of memory (which obviously cannot be infinite for any physical or for that matter, chemical realization) available to them. With just two stacks and appropriate calls to them as a memory, one can do a lot. These 2-stack PDAs are called Linearly Bounded Automata (LBA) and can, in principle, resolve all strings of bounded length in the Chomsky Hierarchy of Languages. They belong in the class of the Turing machines (Harrison, 1978).

The above implies that in addition to computations performed with gates there is an alternative approach to computation which uses non-trivial generalizations of the Boolean gates. This alternative is based on the fact that any computation can be posed as statements of Acceptance/Rejection of a string of symbols representing words in a language. The language will be a member of one of the language classes in the Chomsky hierarchy. This, combined with the knowledge that anything can in principle be represented by a string (Rich, 2008) gives us the possibility of recasting any computational problem soluble with many gates as a collection of problems soluble with fewer but more advanced automata. The automata, however, are more complex than the Boolean gates [An actual example of the latter is provided in a very complex fashion and in ways that still are not fully understood (Searls, 2002, 2012) by natural life and its central use of genetic information].

**Figure 2:**
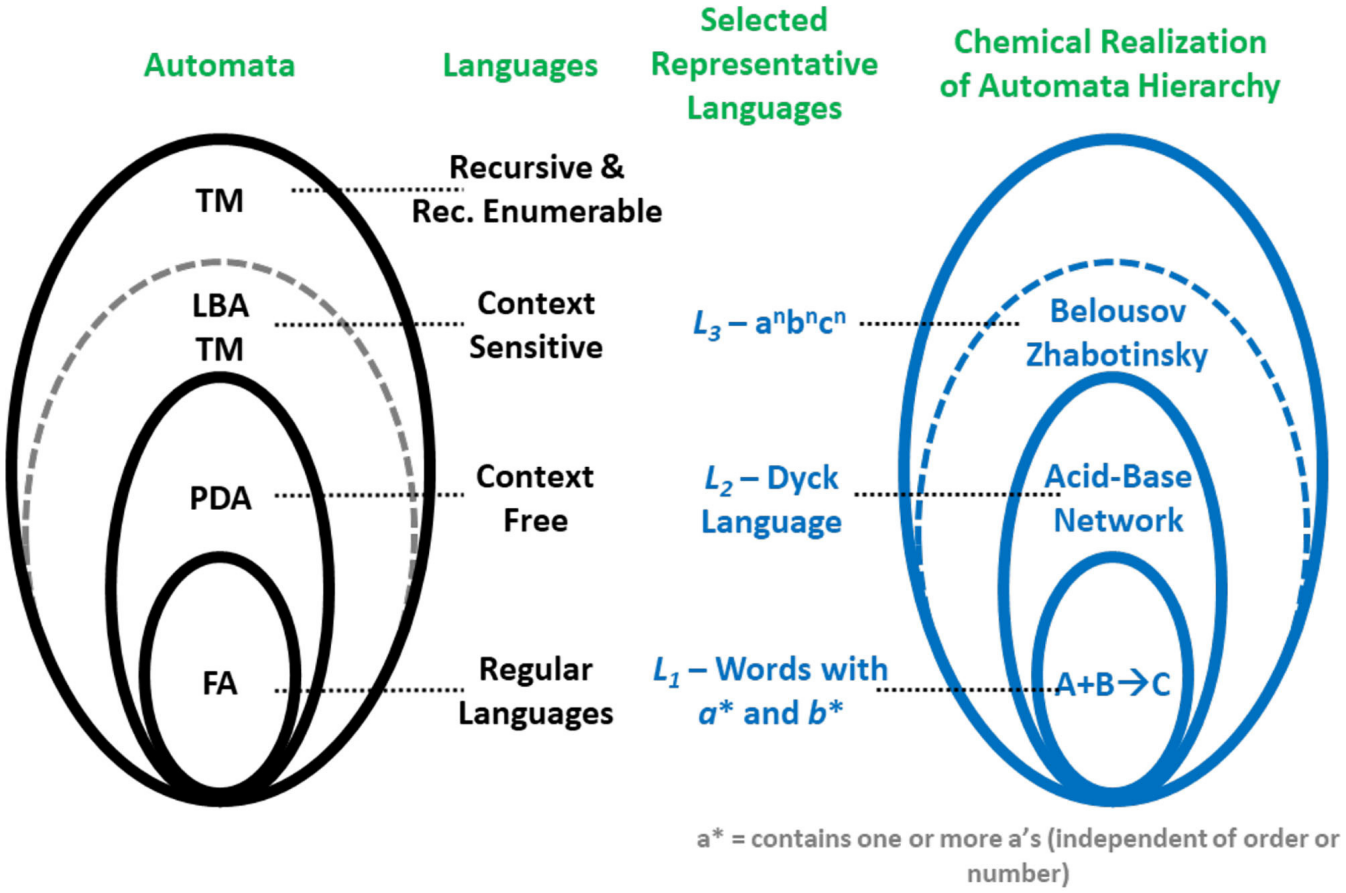
Correspondence between the automata hierarchy, the Chomsky hierarchy of languages on the left, and the experimental realizations of native chemical automata on the right side of the figure. All these hierarchies are inclusive, e.g., the BZ reaction can be shown (Dueñas-Díez and Pérez-Mercader, 2019a, 2020) to recognize not only a context sensitive language (L_3_) but also a context-free language (L_2_) and a regular language (L_1_). Reprinted from Dueñas-Díez and Pérez-Mercader (2020) Copyright (2020) with permission from Nature Scientific Reports.

In the case of chemistry where the use of Boolean gates, even for reasonably error-tolerant computational problems is prohibitive, having access to automata provides a clear advantage. Thus, access to chemical automata then offers the potential to open new horizons for chemical computing. Recent breakthroughs along these lines demonstrate that there exists a direct correspondence between chemical reactions and the automata in the Chomsky hierarchy in Figure 2 (Pérez-Mercader et al., 2017; Dueñas-Díez and Pérez-Mercader, 2019a).

Any materially realizable automaton with a tape is bounded in tape length, as any infinite tape requires an infinite amount of energy to operate. Because of this, the top automaton that can be physically realized with actual matter (chemical or otherwise) is the class of the finite tape-length Turing machines (the LBAs). Furthermore, for an automaton to be configurable and used as a finite tape-length Turing machine the automaton must be capable of handling non-linearity and have access to at least two memory stacks. These requirements are satisfied in chemistry by oscillatory chemical reactions and bring us to the subject of non-linear chemical oscillators (Epstein and Pojman, 1998) which will play a major role in native chemical computation and allow us to take full advantage of the above in chemical computation.

## Oscillatory Chemistry and Native Chemical Computation

As we have seen, to implement the most powerful (physically realizable) automaton we need to have access to non-linearity, for example as provided by a switch, and at least two memory stacks. To translate the above into a chemical computing automaton, we have to ask which chemical reactions offer those two capabilities. If correctly implemented we should then be able to build a chemically operated LBA-Turing machine automaton for the rest of this introductory review, and in the interest of conciseness, we will not consider examples of chemical FA or 1-stack PDAs; for full details on these chemical automata, the interested reader can consult (Pérez-Mercader et al., 2017; Dueñas-Díez and Pérez-Mercader, 2019a,b; Dueñas-Díez and Pérez-Mercader, 2020).

A chemistry where the presence of a switch is essential is oscillatory chemistry: a chemical switch, together with autocatalysis and feedback, is a basic component of the mechanism that underlies oscillatory behavior in chemical reactions (Volkenshtein, 1983; Epstein and Pojman, 1998). We can then think of chemical oscillations as adequate providers of the non-linearity required by fully fledged computation. But, remarkably, chemical oscillations bring with them another feature useful for enabling chemical computation. Indeed, chemical oscillations, like any other oscillatory phenomena, are characterized by two interdependent (which for the particular case of linear oscillators are independent) physical variables: the frequency and the amplitude of oscillations. Furthermore, since the instantaneous state of a chemical reaction taking place in a reactor is the result of adding aliquots of the reacting substances to the reaction, we see that one can think of the frequency and amplitude and their value in the reactor at a given time as directly related to two stacks of memory. These “stacks” are of course dynamically interconnected by the non-linear oscillatory reaction, and their time dependent values are correlated with the internal reaction pathways visited in the course of the reaction. These will change upon addition of reactants or other modifiers of reaction intermediates, such as changing the pH or the concentrations of reaction catalysts in the reactor.

**Figure 3:**
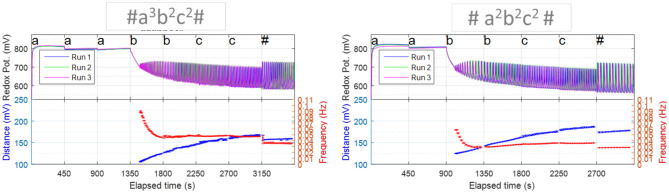
Oscillatory reactions contain the necessary components for computation. These relaxation chemical oscillations not only show the key non-linearity, but also have two stacks, i.e., two oscillatory features (e.g., frequency and amplitude or equivalent measure). Note that the oscillatory features vary depending on the order of the sequence. Each sequence experiment was repeated three times, from whose results the plotted error bars were calculated. Reprinted from Dueñas-Díez and Pérez-Mercader (2019a) Copyright (2019) with permission from Cell Press.

The above indicate that oscillatory chemical reactions contain the necessary components for the native chemical representation of a 2-stack Push-Down Automaton: the 2-stacks and the on-off switch which control the pathways that are visited upon the addition of substrates to the reactor. That is, the oscillatory behavior that would start upon the addition of substrates in the adequate order and concentrations, is the manifestation of the internal state and rules for the operation of the chemical automaton (cf. Figure 3).

The operation of a chemical oscillator brings with it another basic ingredient of chemical computation: aliquots of the substrates and the modifiers of the reaction conditions can be used to translate abstract symbols in a symbolic alphabet. Additionally, the order in which the aliquots are fed to the reactor matters, which is a direct consequence of the non-linear nature of the chemical reaction [non-linear operations do not commute, exp (sin x) is not the same as sin (exp x)]. Therefore, it follows that we can represent meaningful information using aliquots of these substances as the letters of an alphabet. In other words, just as we can represent anything by strings of symbols we can represent information by means of a sequence of aliquots fed to the core, i.e., the chemicals not entering into the above mentioned category of “substrates” and modifiers of an oscillatory reaction.

All the canonical languages and their grammars that enter in the Chomsky hierarchy can be represented by finite size alphabets. And they can be further transformed (without getting out of their level in the hierarchy) by means of some encoding.

The languages that can be handled by oscillatory chemistry are all the languages at the level of the LBA-TM in the Chomsky hierarchy and below (cf. Table 1, The Chomsky hierarchy). These are the Regular Languages (RL), the Context Free Languages (CFL), and the Context Sensitive Languages (CSL).

Useful examples of the simpler languages in the hierarchy are provided by the following languages. A particularly simple RL is the language L_1_ of all words using a two symbol alphabet, a and b, with the words containing at least one instance of symbol a and at least one instance of symbol b. Words in this language would be “ab,” “aab,” “baa,” or “aaabbbb” sequentially fed to the automaton from left to right. This language [like any regular language (Hopcroft et al., 2007)] does not require counting or memory in order to identify its words. Regular languages are recognized by FA and other automata above them in the Chomsky hierarchy.

For a CFL there is the need for counting and a prototype language is L_2_, the language of well-balanced pairs of brackets. The symbols here are “(“ and “)” which brings with it a notion of order: while the sequence “()” is well-balanced, the sequences “)(”or “))” or “((“ are not well-balanced. The words in this language are called Dyck words (Weisstein, 2009), examples of which are “(())” or “(()())” etc. To implement this language, one needs one memory stack, and its accepting automata belong to the 1-stack PDA class or above in the Chomsky hierarchy.

In a CSL the grammar is more structured than for a CFL (note the “S” for sensitive and the “F” for free in the language type) and this requires the presence of two stacks in the automata that can identify CSLs, one more stack than what is necessary in the cases of CFLs. A typical example of a CSL is L_3_, made up of words of the form a^n^b^n^c^n^ which require at least a 3-symbol alphabet. These words are strings containing n a's followed by n b's and then by n c's. A few examples of words in L_3_ are “abc,” “aabbcc,” or “aaaaabbbbbccccc.” The reader can easily check that by grouping letters one can restrict the more complex languages to provide instances of languages with a lower complexity. The physically realizable automata in the Chomsky hierarchy that recognize CSLs are the LBAs. There is no 1-stack PDA or FA that can recognize them.

Of course, to perform a “native chemical computation” the letters in the alphabet themselves must be chemically represented. This is achieved by the translation of the letter symbol say, a, b, or c into an aliquot of a suitably chosen and reacting chemical species. The aliquot corresponding to each letter in the word is sequentially added to a one pot reactor which will process each letter during a fixed timeτ. This time must be long enough so as to get the aliquot thoroughly mixed with the rest of the chemicals already in the reactor.

The result of the computation, that is, whether the input word is or is not in the language for whose recognition the automaton (chemical reaction) was engineered, can be a physico-chemical signature produced by the reaction at the end of the computation. In this context, it is important to notice that, just as in the theory of abstract automata, we need a beginning and end of word symbol, which following the tradition of automata theory we will denote by “#.” For example, in the LBA-Turing machine described in Section **Building a Chemical Turing Machine Using the BZ Reaction Chemistry**, this symbol is chemically translated as an aliquot of the BZ ruthenium catalyst. The # symbol is fed to the automaton before the first and also after the last symbol in the word being processed. After the # is processed the two oscillator variables *D* and *f* (see at the end of this review a list with Nomenclature and Definitions for their definitions) will have specific values. These values reflect the state of the reactor after the preceding symbols have been chemically processed in the course of the computation.

That is, once the aliquots for the symbols and the composition of the rest of the reaction components are formulated, the result of processing any words (in the language accepted by the automaton or not in that language) is represented by the values of the system's state variables at the end of the computation. The values of these variables characterize the relationships between automaton, language, chemical representation of the alphabet, and details of the word processing carried out by the automaton. There is a characteristic and systematic response for the words in the language recognized by the automaton. It is experimentally verified that the locus of these values is a smooth convex upwards curve that connects words in the language and discriminates the words not in the language by dividing the *(D,f)*-plane into two disjoint regions of rejected words. How do we interpret these? What “state variable” can be associated with the sequence recognition process and its physical interpretation? Answering these questions will lead us to identify some profound properties of native chemical computation, its use and about the similarities and some differences between chemical computation and other forms of general computation.

It should not come as a surprise that a chemical automaton will produce correlated values of some physical variable for a computation ending in acceptance. This is so because the result of the computation of a submitted sequence will be either acceptance or rejection, and whereas in general there could be many types of rejection, the acceptance only happens for sequences sharing the pattern (order) of words belonging to the identified language. Since a computation is a process of molecular recognition, we expect that recognition would entail some form of “optimization” or reduction in uncertainty. That is, a reduction in the entropy of the system. Therefore, given an automaton with its chemical recipe for language recognition (including the recipe for the symbols), in chemical space the recognition process will be equivalent to following “paths” in the reaction mechanism for free-energy dissipation during the computation (Landauer, 1961; Notice that this implies that a chemical computation of sufficient complexity can enable, for example, “if-then-else” rules and other basic constructs related to programmability. In this regard, cf. also Landauer, 1961).

**Figure 4:**
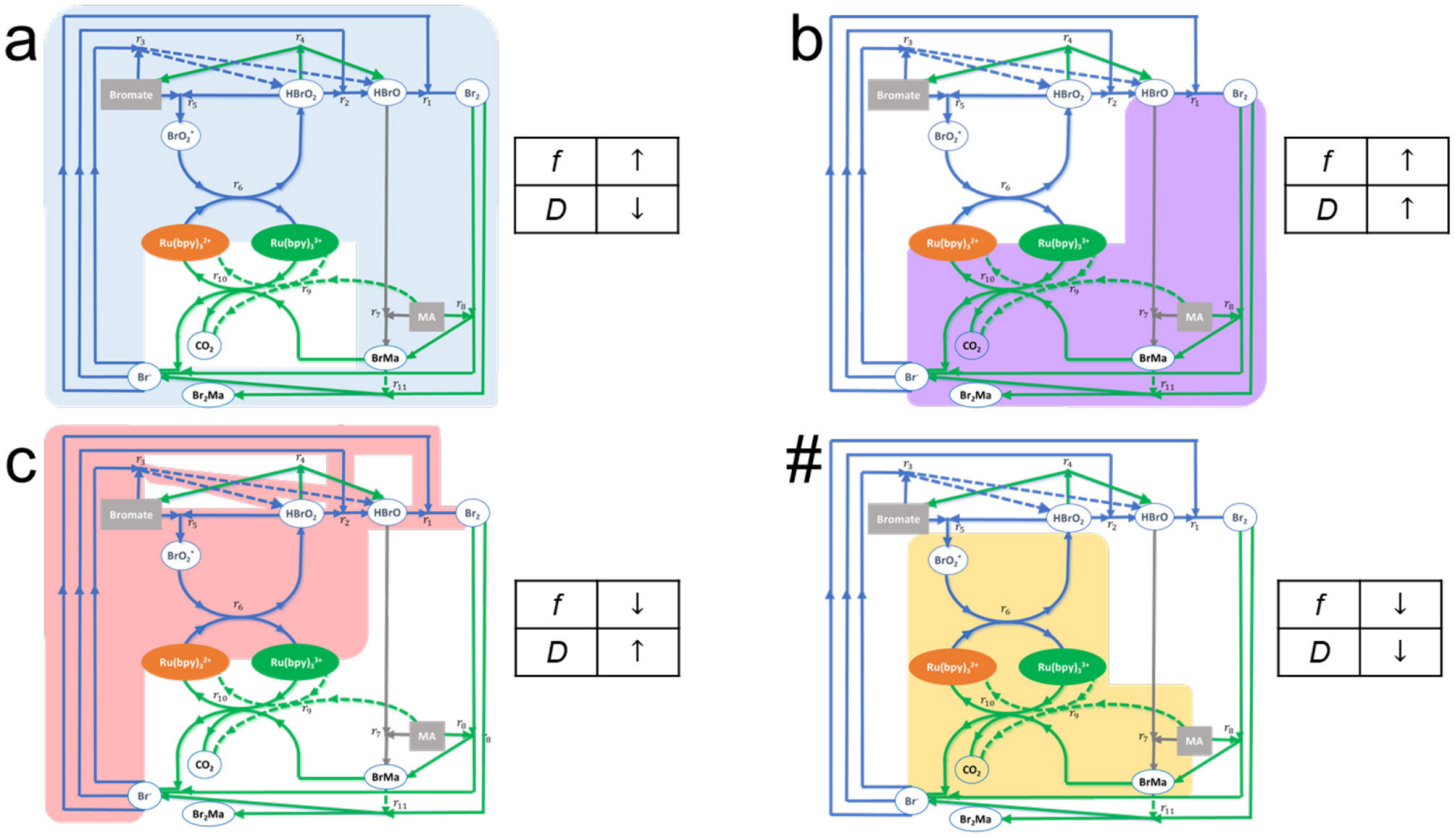
Effects on the dominant pathways in the extended FKN model of the BZ reaction mechanism, as well as on the oscillatory metrics *f* and *D* due to the specific assignment of alphabet symbols in the implementation of a chemical Turing machine built using the BZ oscillatory chemistry, Here the aliquots for the symbols are **(a)** Sodium Bromate, **(b)** Malonic Acid, **(c)** NaOH, and **(#)** Catalyst Ru(II). The colored areas represent the set of reaction pathways affected by each of the alphabet symbols. In the case of **(a,b,#)** the highlighted reactions are enhanced, whereas in the case of **(c)** the highlighted reactions are slowed down. Reprinted from Dueñas-Díez and Pérez-Mercader (2019a). Copyright (2019) with permission from Cell Press.

Thus, we expect that (as is actually observed) for words in the language recognized by the automaton, accepted words, there will be a locus connecting the automaton's outputs for the words once all the symbols in the words have been fully processed. Depending on the actual details of the sequence of letters making up the rejected words, the words will fall into one or the other disjoint areas (subspaces). That is, the particular order of the symbols in the sentence being computed determines the manifold on which they will “land” at the end of the computation. Mathematically this can be related to the non-commutativity of non-linear operations, but chemically this is a manifestation of the fact that each aliquot will excite a particular subset of pathways within the reaction mechanism (Figure 4). In other words, thinking of aliquots as “chemical fuels” for these non-equilibrium systems, the total energy dissipated (or consumed) by the system in recognizing or rejecting words will depend on both the internal reaction pathways excited in a particular order by the chemicals representing the word sequence, and on the total length of time these specific pathways are visited and remain excited. In fact, we can follow the kinetics and roughly trace the pathways within the reaction mechanism that are visited by the substrates in the course of a computation (This, of course, leads to the notion that word recognition by a chemical automaton is associated to some kind of optimization).

For example, in the extended FKN model of the Belousov-Zhabotinsky redox reaction (Field et al., 1972; Dueñas-Díez and Pérez-Mercader, 2019a), Figure 4, we display the “traffic” patterns in the reaction pathways that are generated when each of the species ${\text{BrO}}_{3}^{-}$, MA, Ru catalyst, or H^+^ are added. (In what follows we will be using the stoichiometry and level of chemical mechanism fine-graining described by the FKN model of the BZ reaction). One can then understand how there may exist energy optimized pathways and how certain combinations of the pathways (i.e., sequence of # followed by a's, b's, and c's and ending with #) would be more suitable than others to balance the respective contributions of the oxidized and reduced states of the reaction. These, in turn, are connected to the previously introduced physical variables *D* and *f* which in the case of the TM are stored in the two stacks. For later reference, we note that their respective dimensionalities are inverse time and energy per unit charge as they are a voltage and a frequency.

**Figure 5:**
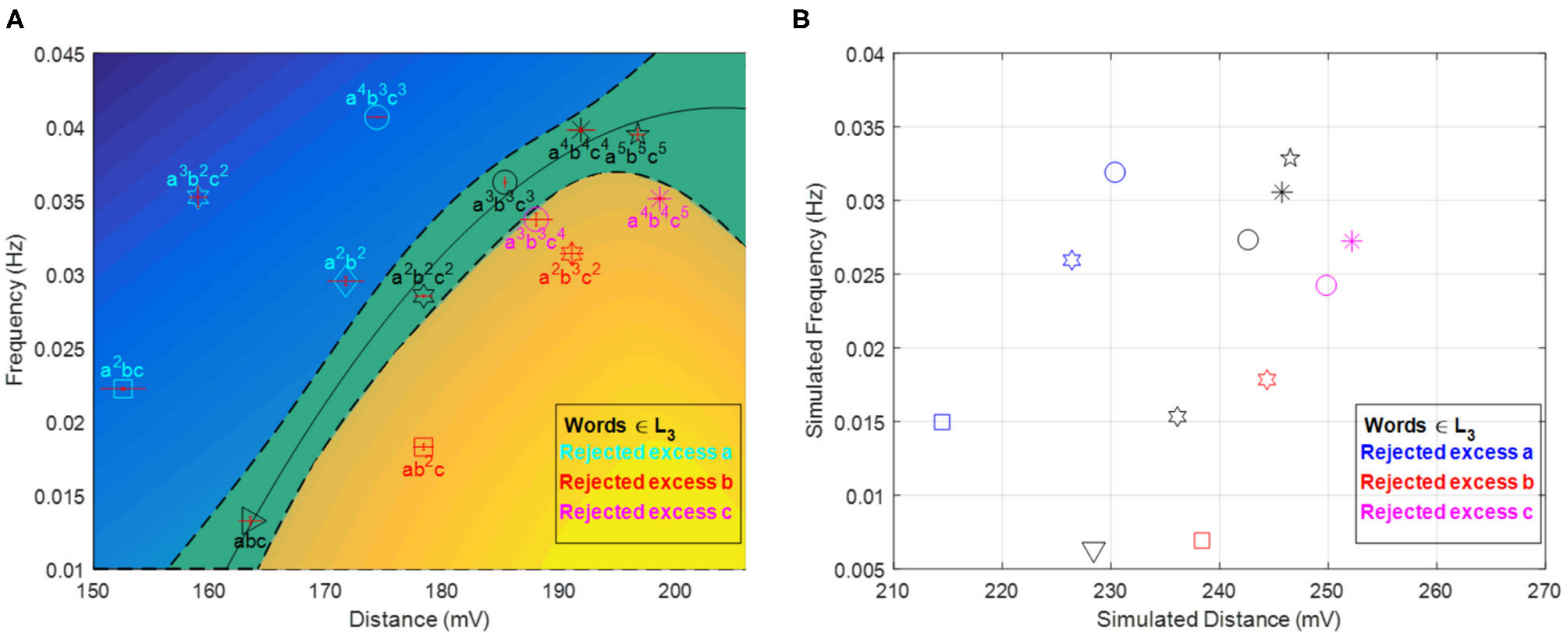
[*D, f* ] plot at the end of computation. Panel **(A)** shows experimental results (each sequence experiment was repeated three times from which the plotted error bars were calculated) while panel **(B)** shows simulation results based on a modified FKN model (cf. Appendices 1 and 2). The locus of accepted words divides the plane in two disjoint regions of rejected words. The relative position of accepted and rejected sequences is reproduced qualitatively by the simulations. Panel **(A)** is Reprinted from Dueñas-Díez and Pérez-Mercader (2019a) Copyright (2019) with permission from Cell Press.

The final values of these variables are at the top of the stacks at the end of the computations. As argued above, their values are remarkably correlated (cf. Figure 5) at the end of the computations for words in the accepted language.

**Figure 6:**
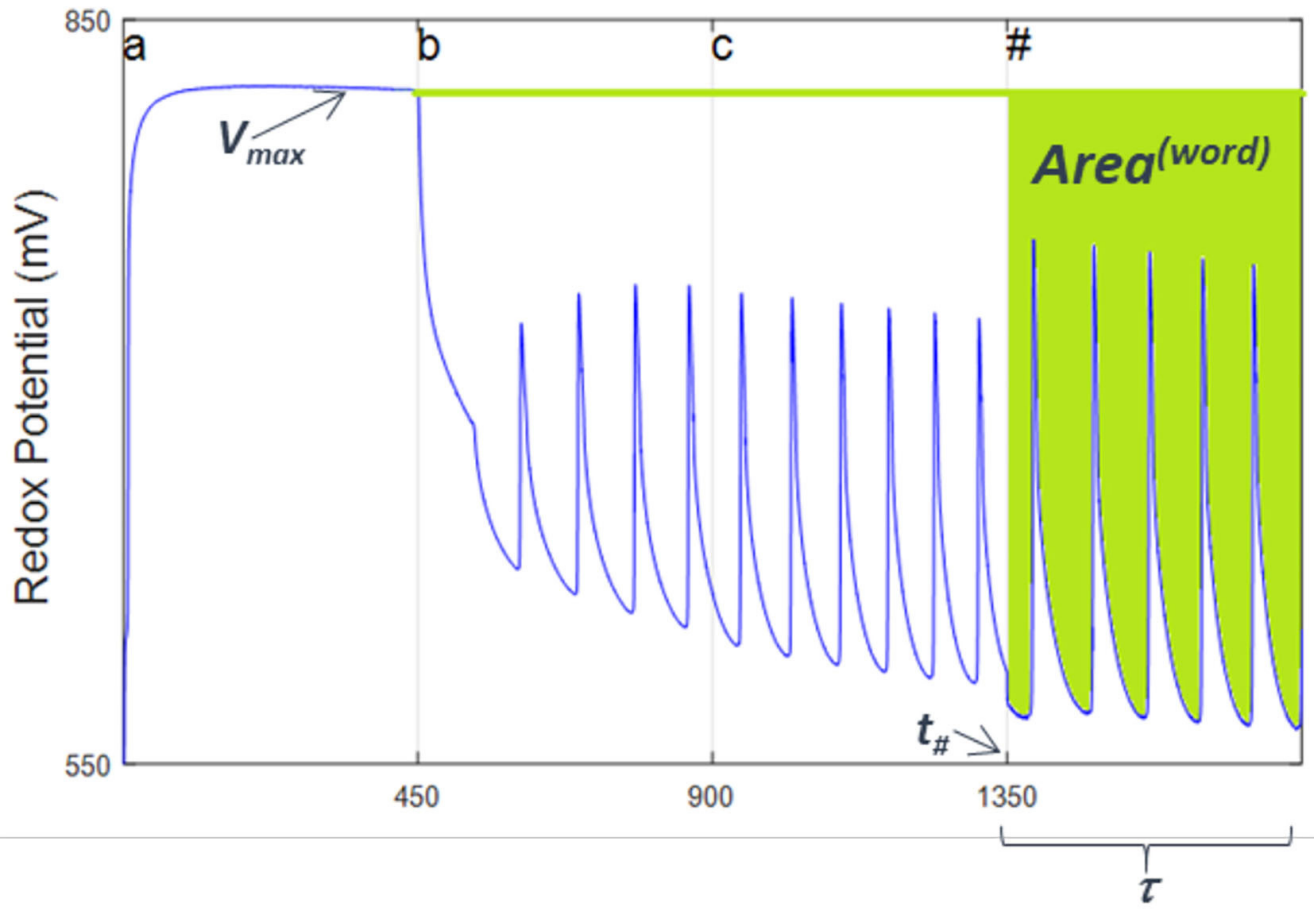
Experimental readout of *Area*^(*word*)^ from the redox potential plot from a given processed word in a native automaton based on Belousov-Zhabotinsky oscillatory chemistry. Shown in this figure is the plot for a word made up by the sequence of one aliquot of **(a)**, followed by one aliquot of **(b)**, then one of **(c)** and finally the end of word symbol, **(#)**. Note how the frequency and amplitude of oscillations vary depending on the sequence, thus in turn determining the value of *Area*^(*word*)^. Time is in seconds. Reprinted from Dueñas-Díez and Pérez-Mercader (2019a) Copyright (2019) with permission from Cell Press.

Of course the values of *D* and *f* have evolved as we added the chemical realization of the symbols (letters) making up the words. This means that they are a function (actually a functional) of the reaction extents, ξ^(*i*)^, of the various reactions that have been visited during the processing of the words. This suggests that instead of looking directly at the values of *D* and *f* , we look at them in a way where the use of the extents is explicit, while we still examine the final state of the reaction (Experimental numerical values for the process can be obtained if we consider the evolution of the reaction redox profile as a function of time). The processing of the beginning-of-sequence and end-of-sequence symbol, #, will give us a read out of the relative weights of the reduced and oxidized extents of reaction at the end of the computation. To implement experimentally the above procedure, we consider in a *V*_*redox*_ vs. *t*_*seq*_ plot the fraction of the initial area that has remained during the computation in the oxidized state after the addition of the first symbol to the Turing machine reactor. This fraction is equal to the area in the *V–t* (voltage vs. time) plane representing the evolution of the redox potential after processing of the first symbol has taken place ~ *V*_*max*_ × *t*, minus the area below the redox voltage spanned by the experimental trace of the *V*_*redox*_ *(t)* curve during the processing of the final # (see Figure 6). Time units are seconds. We will call this difference the processed word's “area,” *A*^(*Word*)^. It is given by

(1) $$\begin{array}{r} \begin{array}{c} A^{\left(Word\right)}\equiv V_{\max}\cdot \left(\tau -30\right)-\int_{t_{\#}+30}^{t_{\#}+\tau }V_{osc}\left(t\right)dt \end{array} \end{array}$$

where *V*_*osc*_ has dimensions of energy divided by electric charge and the 30 s added to the lower limit of the integral are introduced to allow for any potential transients to settle. If multiplied by *e*, the charge of the electron, this quantity [i.e., *e* times *A*^(*word*)^] has the dimensions of the action in physics which, as we will describe below, plays an important role providing a physico-chemical interpretation of a native chemical computation.

Using Nernst's law and a model for the kinetics of the oscillating reaction one can readily compute the integrand of Equation (1) which can then be used (see Section Building a Chemical Turing Machine Using the BZ Reaction Chemistry describing the construction and formulation of the BZ TM) to calculate the values of the area for the word processed by the chemical automaton. Remarkably, one finds that the area for words in the language accepted by the Turing Machine, plotted as a function of the total time it takes to process all the symbols in the word (including the two instances of the end of sequence symbol # at the beginning of the word and at word end), follows a straight line in the *A*^(*Word*)^ vs. *t* plane. We also see that the words not in the language follow different paths which depend on how or when in their symbol sequence they come out of the automaton's accepted language. They are not on the same straight line as the words in the language, which indicates that some (global, internal, and autonomous) optimization takes place during the course of the native chemical computation being carried out by the automaton. Moreover, the slope of this straight line can be made to vanish by suitably adjusting the molarity of the aliquots representing some of the alphabet symbols. This implies^2^ that for words in the language, the quantity *A*^(*Word*)^ does not change (is invariant) under changes in the total time spent by the automata performing the computation (Dueñas-Díez and Pérez-Mercader, 2019a). That is,

(2) $$\begin{array}{r} \begin{array}{c} \delta A^{\left(Word\right)}{|}_{WordinLanguage}=0 \end{array} \end{array}$$

But *A*^(*Word*)^ is a integral over time, and therefore Equation (2) represents at least a piece for the formulation of a variational “principle,” as in the cases of the principle of least action in mechanics (cf. Feynman et al., 1965; Goldstein et al., 2002; Lanczos, 2012) or of entropy generation in chemistry (cf. Prigogine, 1955; Yourgrau and Raw, 1957). Paraphrasing Feynman, we can say that words in the language follow paths in the area-sequence length plane that keep *A*^(*Word*)^ at a constant value.

Let us further explore what is at work behind these observations. From Nernst's law we have that for a redox reaction

(3) $$\begin{array}{r} \begin{array}{c} V_{osc}=V_{0}+\frac{RT}{\nu F}\ln\left(\frac{\left[Ru{\left(bpy\right)}_{3}^{3+}\right]}{\left[Ru{\left(bpy\right)}_{3}^{2+}\right]}\right) \end{array} \end{array}$$

where *V*_*o*_ is a reference voltage $\left[Ru{\left(bpy\right)}_{3}^{3+}\right]$ and $\left[Ru{\left(bpy\right)}_{3}^{2+}\right]$ are the (instantaneous or time dependent) concentrations of catalyst in the oxidized and reduced states, ν is the number of electrons involved in the redox reaction, and *F* is Faraday's constant. Equation (3) multiplied by ν and Faraday's constant, is the Gibbs free-energy *G*(*t*) for the oscillating redox reaction (Kuhn and Försterling, 2000), which inserted into Equation (1) tells us that for isothermal and isobaric conditions

(4) $$\begin{array}{r} \begin{array}{c} \delta A^{\left(Word\right)}=\delta \int_{t_{\#}+30}^{t_{\#}+\tau }G\left(t\right)dt \end{array} \end{array}$$

The function *G*(*t*) has a support restricted to the substances involved in the electrochemical sector of the redox reaction, *Ru*^3+^ and *Ru*^2+^. It is a functional of the extents of reaction, ξ^(*i*)^(*t*), for the reactions *i* involved in the changes in concentrations between the two redox states of the catalyst. In the FKN model of the BZ reaction (Dueñas-Díez and Pérez-Mercader, 2019a; see also Appendices 1 and 2 in this review) we have that

(5) $$\begin{array}{r} \begin{array}{c} \left[Ru{\left(bpy\right)}_{3}^{3+}\right]={\left[Ru{\left(bpy\right)}_{3}^{3+}\right]}_{input}+\xi^{\left(6\right)}-6\xi^{\left(9\right)}-4\xi^{\left(10\right)} \end{array} \end{array}$$

and

(6) $$\begin{array}{r} \begin{array}{c} \left[Ru{\left(bpy\right)}_{3}^{2+}\right]={\left[Ru{\left(bpy\right)}_{3}^{2+}\right]}_{input}-\xi^{\left(6\right)}+6\xi^{\left(9\right)}+4\xi^{\left(10\right)} \end{array} \end{array}$$

From Equations (2)–(5) we see explicitly that the Gibbs free energy can be a function of the form

(7) $$\begin{array}{r} \begin{array}{c} G\left(t\right)\equiv G\left(\xi^{\left(i\right)}\left(t\right),d\xi^{\left(i\right)}/dt;t\right) \end{array} \end{array}$$

And the area variation becomes,

(8) $$\begin{array}{r} \begin{array}{c} \delta A^{\left(Word\right)}=\delta \int_{t_{\#}+30}^{t_{\#}+\tau }G\left(\xi^{\left(i\right)}\left(t\right),\;d\xi^{\left(i\right)}/dt,t\right)dt. \end{array} \end{array}$$

## Interpretation of Word-Acceptance in a Native Chemical Turing Machine

We have seen above that an appropriate phenomenological reformulation of the aliquots for the symbols representing the words in the language accepted by the native chemical Turing Machine reveals three important aspects of the chemical computation. First that for every word not in the language recognized by the TM, the TM automaton classifies them in the amplitude-frequency plane as being either below or above a path in that plane which connects the machine's response points corresponding to words in the language for which it was programmed. Secondly, that one can transform the previous response in the *(D,f)* plane into an equivalent response in an “*A*^(*Word*)^” vs. word-symbol count plane where the words in the language fall in a straight line. And thirdly, that this straight line can be rotated into a horizontal straight line in the *(D,f)* plane by an additional chemical concentration adjustment of the aliquots translating the word in the language of the automaton to new (chemical concentration reformulated) alphabet symbols. It is important to note that these reformulations involve the concentrations but not, necessarily, changing the nature of their molecules [The latter can be shown to be due to some invariance or symmetry in the chemical kinetics (cf. Noether, 1918; Nicolis and Prigogine, 1977; De Groot and Mazur, 2011) which will not be discussed in this review].

Let us concentrate on the third remark above. Put succinctly, it is saying that there is a chemical equivalent of the “action” in physics which can be used to account for the evolution of the state of the chemical automaton as it processes symbols, and that this equivalence is such that (1) gives the same value of the action (area) for any accepted word, (2) this value is independent of the word length so long as it accepted, (3) the distance between consecutive accepted words is a constant, and (4) that after a “rotation” in chemical space, the distance between consecutive accepted words is the shortest possible in a two dimensional space [i.e., the brachistochrone, or “curve down which a bead sliding from rest and accelerated by gravity will slip (without friction) from one point to another in the least time“ (cf. Goldstein et al., 2002) in this action vs. time plane is a straight line].

Indeed, the value of the area after processing the # symbol provides a direct measure of how much the extents of reaction have progressed during their visits to the oxidized and reduced states. That is, this action gives us information on how the changes in the concentrations of Ru^3+^ and Ru^2+^ have evolved during the full course of the computation. Its values and functional evolution clearly involve the Gibbs relationship for entropy production (cf. Baierlein, 1999) with the reaction extents ξ(*t*) (which reflect changes in concentrations as a function of time) as reaction coordinates. It is interesting to note that the constant area/action functional harkens the presence of some optimal dynamical evolution of the BZ chemistry when configured to operate as a TM and fed with words in the language it recognizes. In other words: some physical quantity must be optimal when the TM processes and accepts words in its language.

As motivated earlier in the review, a quantity that can naturally reflect what occurs during a chemical computation is the non-equilibrium entropy which is produced by the “flow” of the *i*-th reaction, *dξ*^(*i*)^/*dt*, driven by its conjugate force, the activity for that reaction *A*^(*i*^^)^ (cf. Aris, 1999; De Groot and Mazur, 2011; Yourgrau et al., 2013). One expects that the events of molecular recognition taking place in a chemical automaton will translate into corresponding out-of-equilibrium changes in the entropy. These cannot be arbitrary and will depend on the sequence of chemicals (word) fed to the automaton for its computation. They are constrained by the entropy balance for an out-of-equilibrium system (cf. Martyushev and Seleznev, 2006; De Groot and Mazur, 2011; Yourgrau et al., 2013; Kondepudi and Prigogine, 2014), given by the equality

(9) $$\begin{array}{c} \frac{\partial s}{\partial t}+\nabla \cdot {\text{J}}_{s}=\sigma \end{array}$$

Here, *s* is the specific entropy, $\frac{\partial s}{\partial t}$ represents the net entropy production, **J**_*s*_ is the flow of entropy across system boundaries, and σ is the entropy production density. At constant pressure and temperature the above becomes

(10) $$\begin{array}{r} \begin{array}{c} \frac{ds}{dt}=\sigma +\frac{1}{T}\frac{dQ}{dt} \end{array} \end{array}$$

Upon using the first law of thermodynamics together with the definition of the Gibbs free energy *G* = *U* + *PV - TS* Equation (10) implies that

(11) $$\begin{array}{r} \begin{array}{c} \sigma =-\frac{1}{T}\frac{dG}{dt}. \end{array} \end{array}$$

In other words, the entropy production in the above conditions is the negative of the change in time of the Gibbs free energy [Furthermore, Equation (11) also says that if d*G/dt* is a minimum, σ is a maximum, and vice versa, that is if σ is a minimum then d*G/dt* is a maximum].

Our *in vitro* experimental measurements (made at constant pressure and temperature) together with Equations (8) and (2) imply that for words in the language accepted by the Turing machine using the realization where the composition of aliquots is such that δ*A*^(*word*)^ = 0 and the distance is the shortest between words in the language,

(12) $$\begin{array}{r} \begin{array}{c} \frac{\partial G}{\partial t}=0 \end{array} \end{array}$$

and (after using the techniques of the calculus of variations) this implies that

(13) $$\begin{array}{r} \begin{array}{c} \frac{\partial G}{\partial \xi }=0 \end{array} \end{array}$$

We conclude from Equation (11) (cf. Yourgrau and Raw, 1957) that when processing those words, the entropy production by the Turing machine is zero. That is,

(14) $$\begin{array}{r} \begin{array}{c} \delta {\sigma |}_{WordinLanguage}=0 \end{array} \end{array}$$

Applied to the results of our experiments concerning the area for accepted and not accepted words, this expression tells us that words in the language for which the Turing Machine was designed, generate a constant entropy in the redox sector of the reaction. In other words, the process of word acceptance in a chemical Turing machine brings its entropy to an extremum.

Since the path joining the words in the [*Sequence Length, A*^(*word*)^]-plane is a horizontal straight line, we can also say that the paths for words in the Turing machine all occur for their minimum separation, i.e., they are processed by the Turing machine following the shortest path. Chemically, these σ = 0 paths are accompanied by the presence of specific quantities of radicals and reaction intermediates produced during the process of word recognition. Since these radicals can be used, for example, to induce RAFT polymerization of amphiphilic block copolymers (ABC) followed by their out-of-equilibrium self-assembly (cf. Bastakoti and Pérez-Mercader, 2017a,b; Hou et al., 2019), we infer that the self-organization and dynamical self-assembly properties of such amphiphiles can be linked to the collective properties of the ABC dissipative self-assembly. The above, in turn implies that these properties can be controlled/programmed by a chemical automaton and in particular by a chemical Turing machine. Such applications showcase interesting and suggestive scenarios where the hybrid analog-digital nature of the computation plays an intriguing and suggestive role. For example, one can think of autonomous programmed dynamical self-assembly of functional materials or supramolecular structures (Pearce and Perez-Mercader, 2020).

The experimental and analytical results presented above can be recast as the statement of a variational principle just as in classical mechanics or in non-equilibrium thermodynamics (cf. Goldstein et al., 2002; Lebon et al., 2008) where the action functional (cf. Goldstein et al., 2002; Lanczos, 2012) integrated along the paths of actual physical motion are extremal, and a minimum in the case of Hamilton's principle. The above translates to our case by simply substituting action for our “area” and the extremal path corresponds to paths that include only words in the language of the Turing machine. Applying Prigogine's result on entropy production in the course of a chemical reaction we then conclude that language recognition in the chemical automaton generates, cf. Equation (11), a minimum amount of entropy if the change of the free energy is a maximum and viceversa.

The above provides an interesting view of acceptance states in chemical Turing machines (and arguably for other automata). Interestingly this extremum can be adjusted in the interest of efficiency by making use of the digital-analog nature of native chemical computation and selecting an appropriate aliquot recipe for the symbols. Theoretically, this adjustment can be done with a precision of a few parts in 10^21−23^ and opens an intriguing set of questions related to the reversibility of computations done by chemical automata.

In summary, we have seen that a native chemical computation at the Turing machine level can be understood in terms of extremal entropy production. This is accompanied by the release of radicals by the TM in specific proportions which can then be used as chemical inputs to subsequent chemical processes and, therefore, enable the fully chemical and autonomous control of complex systems. This we will explore in the following section which is devoted to the specific example of a BZ-based native chemical Turing machine in the LBA-class.

## Building a Chemical Turing Machine Using the BZ Reaction Chemistry

We are now ready to discuss how an actual chemical Turing machine is built using the oscillatory Belousov-Zhabotinsky reaction to recognize CSL L_3_ (Dueñas-Díez and Pérez-Mercader, 2019a). We will discuss the design and operation of the Turing Machine in both batch mode and flow mode, specifically in a continuously stirred tank reactor (CSTR) mode.

### Chemical Turing Machine in a Batch Reactor

First, the alphabet symbols in the language to be recognized need to be assigned to their counterpart chemical species. Language L_3_ has **a**, **b**, **c**, and **#** as symbols. Therefore, four different chemical species need to be identified that affect in distinct ways the dominant pathways in the reaction mechanism, and, in turn, the observed oscillatory features. The assignment can be done empirically, or preferably, guided by the knowledge on the reaction mechanism and kinetics (cf. Dueñas-Díez and Pérez-Mercader, 2019a, and Appendices 1 and 2). As a rule of thumb, the reactants, the catalysts, and the most interconnected intermediates or chemical species interacting with them, are the best candidates for assignment. Here, the reactants sodium bromate and malonic acid were successfully assigned to **a** and **b**, respectively, and sodium hydroxide, a species affecting the most interconnected intermediate H^+^, to **c**. The catalyst was assigned to **#**. The mapping between the symbols and their effects on the oscillatory features (*f* and *D*) and dominant pathways in the FKN model is discussed in the previous reference.

**Figure 7:**
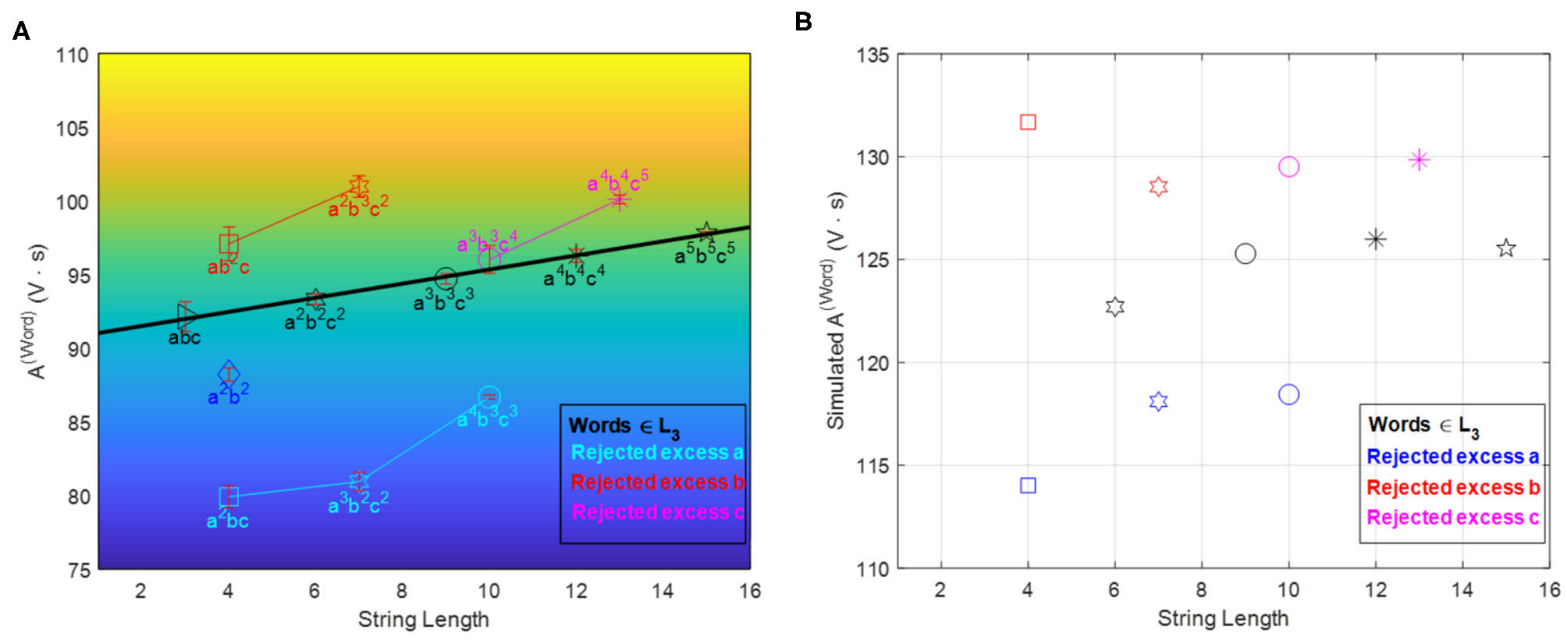
*A*^(*Word*)^ vs. word-symbol count accept/reject criteria. Panel **(A)** shows experimental results (error bars are based on three repetitions of each sequence experiment) while panel **(B)** shows simulation results based on a modified FKN model (cf. Appendices 1 and 2). The locus of accepted words is now linear (in experimental results) and divides the plane in two disjoint regions of rejected words. The relative position of accepted and rejected sequences is reproduced qualitatively by the simulations. Reprinted from Dueñas-Díez and Pérez-Mercader (2019a) Copyright (2019) with permission from Cell Press.

Next, we need to quantify the alphabet recipes and initial conditions that ensure a proper operation envelope of the automaton, i.e., keeping the reaction mixture within the oscillatory regime and simultaneously leading to observable/measurable changes in the oscillatory features whenever a symbol is read and processed. Recipe quantification typically involves optimization, which can be carried out experimentally or aided by kinetic simulations (cf. Appendices 2 and 3). In the first experimental realization, the recipe was optimized experimentally to minimize the gas production that interferes with the redox potential monitoring, and to ensure reliable accept/reject outputs for sequences up to *n* = 5 both in the [*D, f* ] (cf. Figure 5) and *A*^(*word*)^ accept/reject criteria (cf. Figure 7). The time interval between aliquots is a design parameter of the chemical automaton that influences the speed and performance of the machine. It was chosen long enough to ensure that the system gives two full oscillations (after the induction period) once both an **a** and **b** are in the system.

**Figure 8:**
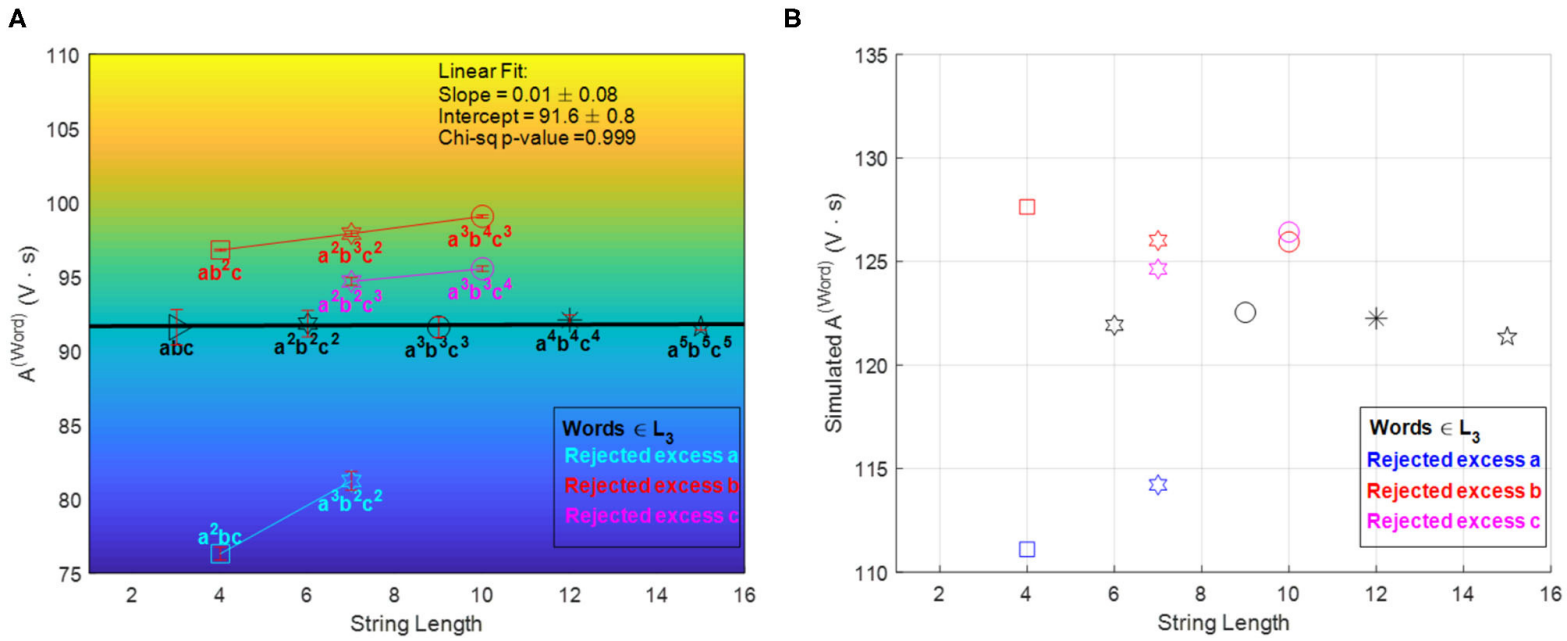
*A*^(*Word*)^ vs. word-symbol count accept/reject criteria after model-based optimization (cf. Appendix 3) and experimental fine tuning. Panel **(A)** shows the experimental results achieving a linear criterion with zero slope with the fine-tuned recipe (error bars are based on three repetitions of each sequence experiment), and panel **(B)** shows the simulation results for the recipe given by the mathematical optimization (prior to fine tuning). The relative position of accepted and rejected sequences is reproduced qualitatively by the simulations. Reprinted from Dueñas-Díez and Pérez-Mercader (2019a) Copyright (2019) with permission from Cell Pres.

The [*D, f* ] accept/reject criterion is non-linear, while the *A*^(*word*)^ shows a linear dependence with respect to the sequence length. Such linearity motivated the further optimization of the recipes to achieve a simpler accept/reject criterion: a constant *A*^(*word*)^ regardless of sequence length (for words in the language). This was implemented using model-based mathematical optimization and a subsequent experimental fine-tuning (cf. Appendix 3 and Figure 8).

The inclusiveness of chemical automata and their reconfigurability have been proven experimentally (cf. Dueñas-Díez and Pérez-Mercader, 2020 and Appendix 4 of this review). Indeed, the batch chemical Turing machine was reconfigured to recognize the context-free language of balanced parentheses L_2_ (Dyck Language) by adjusting the recipe of aliquot **b**. It was also reconfigured to recognize a simple regular language L_1_, the language of all sequences that contain at least one **a** and one **b**. The choice of time interval guarantees adequate performance without requiring further recipe adjustment.

### Extension of Chemical Turing Machine to CSTR Operation

The extension to continuous operation (CSTR) is appealing for several reasons: the autonomous resetting of the machine to process a new input, the potential for faster computations since the machine is already in an oscillating regime at the onset of computation, the availability of a wider range of periodic and aperiodic oscillatory regimes (cf. Hou et al., 2019) to be eligible for accept/reject signatures, and the straightforward ability to reconfigure and to connect several automata such that new computations can be carried out (cf. Cohen, 1991; Hopcroft et al., 2007).

The autonomous resetting of the automaton for running a new input means that the input is “erased” from the reactor after a certain resetting time *T*_*resetting*_ that can be estimated from the residence time distributions (RTD) mean μ_*RTD*_ and standard deviation σ_*RTD*_, which for an ideal CSTR (Levenspiel, 1999):

(15) $$\begin{array}{r} \begin{array}{c} T_{resetting}\sim \mu_{RTD}+2\cdot \sigma_{RTD}\sim 3\cdot \tau_{res} \end{array} \end{array}$$

Which aspects of the design remain the same, and what additional conditions need to be considered when extending native chemical computation in CSTR mode? The choice of alphabet assignment would still be based on the same principles, and the aliquot amount design would follow the same considerations of keeping the system within the oscillatory regime while simultaneously providing a measurable change in the oscillatory features whenever an alphabet symbol is read. The time interval would still be limited by the slowest symbol-pathway pairing, but the effect of flow in reaction needs to be considered as well. The ratio between the resetting time *T*_*resetting*_ and the computational time interval τ influences the maximum length of input sequence that can be processed without erasure of information during the processing of the sequence:

(16) $$\begin{array}{r} \begin{array}{c} l_{\max}\sim \frac{T_{resetting}}{\tau }\sim \frac{3\cdot \tau_{res}}{\tau }. \end{array} \end{array}$$

Hence, τ and τ_*res*_ would need to be carefully chosen to try to maximize the processable sequence length.

The experimental setup for running a native computation in CSTR is more complex than that for a native batch computation, but the only additional required equipment would all be standard to continuous operation.

Another difference with the batch implementation is the initial conditions. In CSTR, the reactor would be in a stationary out-of-equilibrium oscillatory regime before processing the input, and once the sequence is fully processed the oscillations would evolve toward the initial oscillatory features, thus automatically resetting the automaton for a new computation.

Finally, since the machine's operation would start in an oscillatory regime, *V*_*max*_ would never be reached in the CSTR, thus the *A*^(*word*^^)^ criterion needs modification. A possible extension of the previous definition of the area for a batch reactor is

(17) $$\begin{array}{r} \begin{array}{c} A^{\left(word\right)}=\int_{2\;oscillations}V_{CSTR}dt-\int_{2\;oscillations}V_{\#\;interval}dt \end{array} \end{array}$$

where the first term integrates the area below the two last complete oscillations before the beginning-of-sequence # (in the stationary CSTR regime) and the second integral gives the area below the two last complete oscillations within the time interval corresponding to the end-of-sequence #.

## Conclusions

In this paper, we have reviewed recent work on native chemical computation: computations performed exclusively by the molecules involved in chemical reactions without involving constructs external to the chemical reaction mechanism. Oscillatory reactions turn out to play a central role in this area of computation.

A computation is a process in which some available information is input, systematically (“mechanically”) transformed in prescribed ways and output into a form suitable for the implementation of some function. Computations are carried out by computing automata. Abstractly, these are machines that do some computations. Some of these abstract automata require access to infinite lengths of tape for their operation and cannot therefore be practically realized. For the practically realizable automata, both a conceptual and technical parallel can be established with the way in which chemical reactions occur. The information to be processed can be transcribed into aliquots of the reactants for a chemical reaction, translated (transformed) by the reaction and output as the molecular products of the reaction or as the values of some physico-chemical quantities which include entropy, redox potential, or pH. Thus, chemical reactions can be viewed as a kind of chemical automata which operate on information represented with chemistry.

Computation with chemistry is central to biology enabling the beautiful phenomena of life, from the synthesis of proteins, to the life cycle of bacteria, to the workings of the human brain. During the last 50 years or so, many workers (Adamatzky and Costello, 2002; Adamatzky, 2019) have worked on the implementation of computation with chemistry but whenever automata were used in laboratory realizations, they used only a few Boolean gates and, due to the intrinsically noisy nature of the transport of information in chemical form, the computations relied on external constructs to take advantage of effects induced by molecular transport, such as waves or externally made shapes to guide the appropriate chemicals during the computation. With native chemical computation, the information processing takes place within the reaction itself making available for the computation the full reaction mechanism. Not surprisingly, this enables the application of chemical computation to more complex problems. These additional potential applications include the interconnection of automata into networks or architectures such as chemical perceptrons and neural networks which, due to the access of chemistry to more complex automata in the various layers of these neural processors, can see enhancements of their capabilities and the nature of the problems that can be tackled with them when using native chemical computation and other forms of chemical computation or their combination.

It is a well-established notion in computer science that any computing problem can be cast as a sequence of language recognition problems. Computer languages can be classified according to their complexity into an inclusive hierarchy known as the Chomsky hierarchy. In parallel with the language hierarchy there is a hierarchy of automata; that is automata capable of recognizing languages according to their complexity. It can be seen that for the case of chemical computation there is a parallel correspondence of computing automata with classes of chemical reactions. At the top of the materially implementable (i.e., not requiring infinite amounts of energy for their operations) are the Linear Bounded Automata (LBA) which correspond to bounded (finite) tape length Turing machines. These automata require their memory to hold at least two variables and are represented by (non-linear) oscillatory chemical reactions, such as the Belousov-Zhabotinsky reaction, where the two variables correspond to the frequency and amplitude of the redox oscillations. These two observables are non-linearly interrelated in a way that depends on the nature and order of the sequence of reactants being fed to the reaction. The reaction recipe can be formulated so as to recognize languages that only LBAs and automata at their level in the Chomsky hierarchy can recognize.

We have also seen how these LBAs can be reprogrammed and how they recognize other languages below their level in the hierarchy. This opens a window for the simulation of chemistry with chemistry, i.e., without any intermediary translation (cf. Feynman, 1982) and brings us closer to attempt unlocking the power of 10^23^ processor per mol, but whose effectiveness is seriously hindered by the very strong correlation among the computing molecules in the bulk reaction solution at room temperature.

The results of the computation, for example, in the case of the BZ-finite tape length Turing machine, are in the form of radicals, relative concentrations of reaction products, and physico chemical signatures. These can be used to connect with other automata down the line and enable more complex operations, or to use in other chemical processes such as in polymerization reactions (Washington et al., 1999) and even enable the chemically controlled, out-of-equilibrium synthesis of complex molecules from small molecules to self-assembled amphiphilic structures, such as micelles or vesicles (Bastakoti and Pérez-Mercader, 2017a,b). The physico-chemical signatures associated with language acceptance offer an experimental glimpse into the implications of native chemical computing for the implementation of the predictions of the Maximum Entropy Production Principle and its connection with the efficiency of computing processes.

Many questions remain open: from the extension of the above framework to other oscillating reactions, to their application to “computer controlled” molecular and supramolecular architectural assembly from the subnanometer to the micrometer scales, to the study of their implications in non-equilibrium thermodynamics and efficient computation, their direct use in the simulation of chemistry by chemistry, including the development of such processors or their use in the construction of mimics of life without using biochemistry.

## Author Contributions

MD-D wrote the BZ Turing machine section, the appendices and made all the figures. JP-M wrote the rest of the manuscript and created the outline. All authors contributed to the article and approved the submitted version.

## Conflict of Interest

The authors declare that this study received funding from Repsol, S.A. The funder was not involved in the study design, collection, analysis, interpretation of data, the writing of this article or the decision to submit it for publication.

## Footnotes

^1^An “automaton” is an abstract computing machine. Its plural is “automata”.

^2^It also implies that the two-dimensional distance between points in the area vs. time plane that represent words in the language is the shortest (minimum) distance.

## Nomenclature and Definitions

**a:** alphabet symbol in abstract language (in the Chomsky hierarchy). For the experimental Turing machine recognizing *L*_3_, this symbol is transcribed by an aliquot of sodium bromate.
**b:** alphabet symbol in abstract language (in the Chomsky hierarchy). For the experimental Turing machine recognizing *L*_3_, this symbol is transcribed by an aliquot of malonic acid.
**c:** alphabet symbol in abstract language (in the Chomsky hierarchy). For the experimental Turing machine recognizing *L*_3_, this symbol is transcribed by an aliquot of sodium hydroxide.
**A**^(**word**)^: Thermodynamic accept-reject metric, based on integrating in the redox potential vs. time plane, defined as: $A^{\left(Word\right)}\equiv V_{\max}\cdot \left(\tau -30\right)-\overset{t_{\#}+\tau }{\underset{t_{\#}+30}{\int }}V_{osc}\left(t\right)dt$. Times are in seconds.
***D:***The (oscillation) distance *D* between the maximum redox potential value *V*_max_ and the redox potential value corresponding to the center of a given oscillation:

$$\begin{array}{l} D=V_{\max}-\left(V_{T}+\frac{V_{P}-V_{T}}{2}\right) \end{array}$$

***D***_**#**_**:** Value of *D* corresponding to the last oscillation when one time interval has elapsed after the end-# was added.
***f*:** The (oscillation) frequency *f*, as the inverse of the period. The period is taken to be the time elapsed between two consecutive peaks.
***f***_**#**_**:** Value of *D* corresponding to the last oscillation once one time interval has elapsed after the end-# was added.
**F**: Faraday Constant.
**G**(**t**):Gibbs free energy.
**Q**: Heat.
**R**: Universal Gas Constant.
$\left[Ru{\left(bpy\right)}_{3}^{3+}\right]:$ Molar Concentration of oxidized catalyst [Ru(III)].
${\left[Ru{\left(bpy\right)}_{3}^{2+}\right]}_{input}:$ Total Molar Concentration of oxidized catalyst [Ru(III)] added with the input.
$\left[Ru{\left(bpy\right)}_{3}^{2+}\right]:$ Molar Concentration of reduced catalyst [Ru(II)].
${\left[Ru{\left(bpy\right)}_{3}^{2+}\right]}_{input}:$ Total Molar Concentration of oxidized catalyst [Ru(II)] added with the input. **s**: Specific Entropy.

**T**: Temperature.

**T**_**resetting**_: Resetting time in chemical automata in CSTR mode. Time needed to erase a computation and set back to the steady oscillations.

**V**_**max**_: Maximum redox potential value, when all catalyst is in the oxidized form.

**V**_**P**_**−V**_**T**_: Trough-to-peak amplitude of the redox oscillation.

**V**_**T**_**:** Redox potential value of the trough of the oscillation.

#: beginning and end-of-sequence symbol. For the experimental Turing machine recognizing *L*_3_, this symbol is transduced by an aliquot of ruthenium(II) catalyst.

**μ**_**RTD**_**:** Residence time distribution mean (CSTR operation).

**ν**: Number of electrons exchanged in the redox reaction.

**ξ**^(**i**)^: Reaction extent corresponding to reaction *i* in the FKN kinetic mechanism of the Belousov-Zhabotinsky reaction.

**σ**: Entropy production density.

**σ**_**RTD**_:Residence time distribution standard deviation (CSTR operation).

**τ****:**Time Interval between aliquots in native chemical automata.

**τ**_**res**_: Nominal Residence time (in CSTR operation).
