# Supplementary material for: Native Chemical Computation. A Generic Application of Oscillating Chemistry Illustrated With the Belousov-Zhabotinsky Reaction. A Review
Source: Front Chem. 2021 May 11;9:611120. doi: 10.3389/fchem.2021.611120 (PMC8144498; doi:10.3389/fchem.2021.611120)
Supplement: Supplementary file 1 [file Data_Sheet_1.docx]

**APENDICES**

# Appendix 1 – Computer Simulations and FKN model

A representative reaction mechanism and kinetics greatly facilitates the design and implementation of a chemical automaton, saving time and effort. Hence, mechanistic information and its computer simulations can be used:

(1) To identify the chemical alphabet and determine how each alphabet symbol affects the output, i.e., to identify the distinct input-output signature for each of the chemical alphabet symbols.

(2) To quantify the aliquot’s strength for each symbol such that sufficiently long input sequences can be processed by the automaton, while ensuring that the automaton’s output can be reliably read/measured.

(3) To elucidate the chemical counterpart of all elements in the abstract automaton description (e.g. septuple in TM etc).

(4) To optimize the performance of the chemical automaton, by e.g. optimizing the alphabet aliquots to achieve an optimal goal or enhance the automaton’s performance.

Points 1, 2 and 4 are eventually fine-tuned experimentally, but by using mechanistic information and computer simulations, the number of experiments required is greatly reduced, and the implementation thus accelerated.

The Belousov-Zhabotinsky chemical reaction has been extensively studied and there is currently a variety of reaction mechanism and kinetic models available in the literature. For the design and implementation of BZ-based chemical automata the model was chosen based on the following criteria:

(1) The model simulations provide as realistic as possible oscillatory features, induction period, decay trends, and predictions of the oscillatory envelope.

(2) The model is simple enough to be coupled to optimization algorithms. Unnecessarily complicated kinetic models would slow down considerably the optimization runs.

Taking this into consideration the simplest, the Oregonator model, would fall short while the Gao-Försterling model would be too complicated, so we finally used the well-known Field-Köros-Noyes model (for details on this and the material in this Appendix, the interested reader is invited to consult Dueñas-Díez and Pérez-Mercader, 2019a, SI Section 4).

This mechanism simulated correctly the automata operated dynamics, except in cases with too large bromate concentration, in which the model would predict that the system crosses a bifurcation leaving the oscillatory regime while in practice the system still oscillates. The model was thus modified, inspired by the more detailed malonic acid bromination kinetics in Gao-Försterling, extending the model with the following:

$$\begin{matrix} {CHBr(COOH)}_{2}{+ Br}_{2}\to{C\mathrm{Br}_{2}(COOH)}_{2}+ \mathrm{Br}^{-}+ H^{+} & r_{11} \end{matrix}$$

Also, since one of the most connected intermediates in the reaction mechanism is $H^{+}$, and we wanted to explore an acid or a base as a potential alphabet symbol, we extended the model by including the acid-base neutralization reaction (cf. Dueñas-Díez and Pérez-Mercader, 2019a, SI Section 4):

$$\begin{matrix} \mathrm{OH}^{-}+ H^{+}\to H_{2}O & r_{12} \end{matrix}$$

Since the proton concentration is in large excess with respect to the potential amount of NaOH added with the aliquot, the model assumes all $\mathrm{OH}^{-}$ is instantaneously and completely consumed, decreasing the proton concentration $H^{+}$ and thus affecting all the rate constants that depend on the proton concentration $H^{+}$.

# Appendix 2 – Model Implementation

The modified version of FKN model was implemented in Matlab®, and simulations run on a laptop with an Intel® Core™ i7-7820HQ CPU @2.90 GH processor and Windows 64 Bits Operating System.

All the kinetic parameters, initial conditions, and automata operation parameters are reported in (Dueñas-Díez and Pérez-Mercader, 2019a, SI section 4). Note that the kinetic parameter for reaction $r_{11}$ was estimated from the Gao-Försterling model, and to simulate the redox potential E (in V) from the catalyst concentrations $x_{3}= \left[ {\mathrm{Ru}\left( \mathrm{bpy} \right)}_{3}^{3+} \right]$ and $x_{10}=\left[ {\mathrm{Ru}\left( \mathrm{bpy} \right)}_{3}^{2+} \right]$, Nernst equation was used .

The addition of a symbol to the machine every 450s is directly simulated as an instantaneous step change of the concentrations of the corresponding chemical (i.e. bromate concentration if **a**, malonic acid if **b,** proton concentration if **c**).

The processing of an input is thus simulated by a sequence of 3n+1 consecutive simulations of the ODE system, each one simulating the dynamics of the system during one time interval, where the initial conditions are the final value of the variables of the previous time interval plus the step change in concentration of the corresponding chemical to the current time interval. For example, for simulating ‘aabbcc’, 7 consecutive simulations of the system of ordinary differential equations (ODEs) is needed.

# Appendix 3 – Optimization

In the experimental realization of automata, optimization may be typically needed to:

(1) Find the language alphabet recipes that ensure a proper operation envelope of the automaton.

(2) Enhance the automaton’s operation, i.e. improve the accept/reject criteria.

(3) Reprogram the automaton to recognize another language.

The optimization can be carried out purely experimentally or, preferably, by a combination of simulation-based mathematical optimization and experiments.

In the BZ-based chemical Turing machine recognizing L_3_={ a^n^b^n^c^n^ for n>0} in (Dueñas-Díez and Pérez-Mercader, 2019a), recipes for the alphabet symbols were chosen to minimize the amount of gas (CO_2_) produced during the reaction, since gas interferes with the redox metering, giving noisy redox potential plots, which in turn increases the errors in the metrics used for the accept/reject criteria. As shown by equations $r_{9}$ and $r_{10}$ in the modified FKN mechanism (Dueñas-Díez and Pérez-Mercader, 2019a, SI section 4), the alphabet symbol influencing the most the gas production is malonic acid (symbol **b**). Hence, the **b** recipe was the variable to be optimized, and the optimization objective was to achieve low-noise redox potential plots (in particular for the time interval corresponding to end-of-sequence **#**) for words with n in the range 1 to 5, both included. The **b** recipe was successfully tuned experimentally to 0.5 ml of a 3.5 M stock solution.

Another optimization of the recipe was also discussed for this BZ-based chemical Turing machine recognizing L_3_, to facilitate the accept/reject interpretation based on the *A^(word)^* criterion, thus enhancing the automaton’s performance. With the recipe based on minimization of CO_2_ production, *A^(word)^* for words in L_3_ showed a linear dependence with the word length. We hypothesized that by reprograming the alphabet aliquots we could obtain a constant *A^(word)^* for words in L_3_, thus simplifying the accept/reject criterion. A model-based mathematical optimization was used to test this hypothesis. First, simulation showed that the most sensitive and adequate symbol recipe to reprogram was bromate (aliquot **a**). Then, the objective function was chosen as (Dueñas-Díez and Pérez-Mercader, 2019a, SI Section 6):

$$F=\sum_{i=2}^{5} \left( A_{i}^{(word)}-A_{n=2}^{(word)} \right)^{2}$$

where $A_{i}^{(word)}$ is the area for word with n=*i* and $A_{n=2}^{(word)}$ is the area corresponding to a^2^b^2^c^2^. The minimum of $F$ is zero, implying that all $A_{i}^{(word)}$ have the same constant value. Otherwise, $F>0.$ The algorithm thus search for the recipe of aliquot **a** that leads to $F=0$ (minimum). We chose a nonlinear gradient-free optimization method, the Nelder-Mead algorithm (fminsearch solver in Matlab®; Nelder and Mead, 1965), since the FKN model is highly nonlinear and may contain discontinuities. The algorithm iterates on **a**, for each iteration it simulates the word sequences in language for n=2,3,4 and 5, evaluates the values of $A_{i}^{(word)}$ for such sequences, and the value of $F$. Depending on the simulated value of $F$ , either applies the rules of the algorithm to get a new estimate of **a** or terminates. The algorithm provided an optimal **a** value of 2.25 ml of a 2M stock solution. This value was then successfully fine-tuned experimentally to 2.40 ml of 2M stock solution.

# Appendix 4 – Inclusive hierarchy

Native chemical automata can recognize languages at its level and lower levels of the Chomsky hierarchy, thus conforming an inclusive hierarchy (cf. Dueñas-Díez and Pérez-Mercader, 2020), in complete agreement with abstract automata theory. Hence, the BZ-based Turing machine, originally designed to recognize context-sensitive L_3_, can be reconfigured to recognize the simpler regular language L_1_ and context-free L_2_.

A reprograming of the BZ-based Turing machine to recognize other languages starts by revising the assignment of chemical species to alphabet symbols. For example, to reconfigure to recognize L**_2_**, whose alphabet symbols are **(** and **)**, it is natural to assign them sodium bromate and malonic acid, respectively, since they are key reactants in Belousov-Zhabotinsky. As a rule of thumb, it is most effective to assign the key reactants first followed by key intermediates or chemical species affecting key chemical intermediates (e.g. the **c ≡** Sodium hydroxide in L_3_). The beginning and end-of-sequence symbol # is again assigned to Ruthenium (II) catalyst. Next step in the reconfiguration is readjusting the symbol aliquots. If the aliquots of **(** and **)** would be kept exactly as in the L_3_ configuration, there would a lot of gas produced during the end-of-sequence **#** that would perturb the redox potential readings, thus perturbing the accept/reject output. In L**_3_**, the “c”s slowed down the reaction, and consequently the gas production. As reported in appendix 3, the malonic acid aliquot, in this case **)**, is the most adequate to tune to avoid excessive gas, and was optimized experimentally to 0.343 ml of 3.5 M (cf. Dueñas-Díez and Pérez-Mercader, 2020).

Now, to reprogram the BZ-based Turing machine to recognize L_1_, we can keep the assignment of sodium bromate and malonic acid to **a** and **b**, respectively. In this case there is no need to readjust the aliquots because the time interval (same used in the three implementations) was already chosen to ensure the onset of oscillation within the time interval once an **a** and a **b** are present in the solution. Note, however, that if one wished to optimize the automaton performance and speed computations, e.g. by reducing the time interval, one could e.g. increase the **a** aliquot to reduce the induction period.
